# Tackling Vaccine Hesitancy and Increasing Vaccine Willingness Among Parents of Unvaccinated Children in Austria

**DOI:** 10.3389/ijph.2023.1606042

**Published:** 2023-08-28

**Authors:** Christian Lenart, Marlene Prager, Marlene Sachs, Christoph Steininger, Charlene Fernandes, Jakob Thannesberger

**Affiliations:** ^1^ Division of Infectious Diseases, Department of Medicine 1, Medical University of Vienna, Vienna, Austria; ^2^ Department of Emergency Medicine, Clinic Hietzing, Vienna, Austria; ^3^ Department of Emergency Medicine, Clinic Donaustadt, Vienna, Austria; ^4^ Department of Internal Medicine, Aga Khan University, Nairobi, Kenya

**Keywords:** COVID-19, vaccine hesitancy, pandemic, intervention strategies, COVID-19 vaccination

## Abstract

**Objectives:** In autumn 2021, there was a surge of COVID-19 infections in Austria, and vaccination coverage stagnated at a below-average level compared to the rest of Europe. Surveys showed that both children and adolescents were the main drivers of the rising infection rates and that vaccination numbers were particularly low in this age group. This was due to widespread vaccination skepticism and hesitancy among parents of unvaccinated children and adolescents.

**Methods:** Here, we describe a novel intervention concept that allowed us to efficiently tackle parental vaccine hesitancy. We designed an intervention series that followed a reproducible format based on online face-to-face seminars in groups of a maximum of twenty people. Each seminar included an anonymous online questionnaire for internal quality control. Moreover, we assessed the motives of parental vaccine hesitancy and asked participants to rate subjective vaccine willingness for their children on a scale of zero to ten.

**Results:** Within 8 weeks, more than 580 people participated in the seminar series. We found that concerns about the side effects of the vaccine were the predominant motive of vaccination hesitancy among the study population. Overall, the intervention could successfully increase the median parental vaccination willingness of participants from a score of five to eight. We identified tree hesitancy motives (distrust towards the pharmaceutical industry, the government, or feelings of restriction from personal freedom) that were associated with below-average vaccination willingness and significant lower increase.

**Conclusion:** With this study we analyzed motives driving COVID-19 vaccination hesitancy among parents of unvaccinated children and reasons of parents to restrain their children from getting vaccinated. The intervention method described here, could effectively address individual concerns on a personal level while at the same time reach a large number of people across geographical and language barriers. Thereby we could significantly increase subjective vaccination willingness of the participants. Our approach is easy to apply, highly cost-effective, and can be used to tackle any kind of medical misinformation.

## Introduction

Vaccines are recognized as the most successful, scientific achievement for the prevention of infectious diseases. Throughout history, vaccines have led to a massive reduction in the incidence of some infectious diseases such as smallpox, measles, polio, and tetanus, to name just a few. With the advancement of highly effective vaccines, the course of the fast-emerging Coronavirus Disease (COVID-19) pandemic has changed significantly. In regions with high vaccine-induced immunity to SARS-CoV2 infection, both incidence numbers and cases of severe COVID-19 illness have decreased dramatically [1]. Until March 2023, more than 13 billion vaccine doses have been administered worldwide [2]. Ever since the initial approval of COVID-19 vaccines in December 2020, multiple studies have consistently shown that the vaccines authorized for use in Europe and the United States have a favorable safety and efficacy profile [3, 4].

Shortly after COVID-19 vaccines were made available, vaccine supply could not keep pace with the growing public demand in most western countries [5]. Increased public demand for COVID-19 vaccination led to systemic vaccination programs in most countries of Europe and the Americas. Public discussions on safety of COVID-19 vaccines revealed similar societal behavior and despite all efforts to make access to vaccination convenient and easy, most countries of the so called western world witnessed a plateau of vaccination rates at around 60%–80% percent [2].

Within the western European countries realm, Austria is amongst the countries with the lowest average vaccination rates at the time of processing this intervention (December 2021). After witnessing a steep rise in vaccination numbers shortly after the initiation of the COVID-19 vaccination program, the demand stalled at about 77% of the Austrian population who had received their first COVID-19 vaccination dose [6]. As the country witnessed a disproportionally high number of COVID cases during the autumn of 2021, the Austrian government launched campaigns aimed at promoting COVID-19 vaccination. Different approaches were conceived and executed, such as easing access to vaccination by opening vaccination counters in supermarkets, airports and even churches. People who agreed to receive their first dose of COVID-19 vaccination were entitled to monetary benefits and enrollment into vaccination lotteries with prizes such as cars and houses. Unfortunately, none of these measures significantly increased vaccination willingness [7]. While the vaccination curve flattened, Austria witnessed a dramatical surge of cases due to the rapid spread of delta variant in the autumn of 2021. Incidence peaked at a rate of 1098.69 per 100,000 inhabitants, putting Austria at the top of the global incidence list [8, 9]. In November 2021, the government decided to employ a lock down for unvaccinated individuals. The measures were subsequently intensified, leading Austria to become one of the first countries to implement a vaccination mandate for all residents aged 18 years and above in May 2022 [10]. However, incidence rates increased later in 2022 with an even higher peak rate of 3598.37 although less severe cases due to SARS-CoV2 variants.

Epidemiologic analysis identified that the uprise of the delta variant wave was predominantly carried by unvaccinated adolescent persons [11]. Therefore, the European medical agency (EMA) granted licensing for COVID-19 vaccination initially for adolescents within the ages of 12–15 years, and several months later, for children aged between 5–11 years. Despite this, these age groups currently had the highest rate of unvaccinated individuals. Hence, soon after approval by the ECDC, the Austrian health authorities adapted their official statement, recommending COVID-19 vaccination for all children and adolescents starting from the age of 5 years [12].

Public and media discussions were strongly biased regarding different motivations and political agendas. The population in general and parents in particular were confronted with opposing opinions of experts and self-proclaimed experts which led to massive uncertainty. Government information campaigns focused mostly on vaccination of the adult population. Parents were advised to discuss questions concerning COVID-19 vaccination of their children with their attending pediatrician or general practitioner. However, medical personnel resources were generally scarce, especially during the corona virus pandemic. As a result, parents felt left in the dark with their questions and concerns which in turn negatively contributed to vaccination rates.

As defined by the WHO Strategic Advisory Group of Experts (SAGE) on Immunization, vaccine hesitancy refers to a delay in acceptance or refusal of vaccination despite availability of vaccination services [13]. Despite this definition, the term is used in a wide range of different interpretations. Thus, vaccine hesitancy describes a heterogenous group of people including persons that feel scared about specific vaccines or specific side effects, people who reject vaccination in principle and people who reject all of forms of modern medical science [13, 14].

Previous research has demonstrated that the diversity of reasons behind vaccination hesitancy result in significant variability in the effectiveness of efforts to enhance vaccination coverage [14–16]. Possible interventions include media advertisement, engagement of influential leaders to promote vaccination or different forms of education initiatives [16]. While education initiatives in general were most successful at changing attitudes, face to face conversations that include narrative aspects were amongst the most efficient means to tackle vaccine hesitancy [16–18]. Taking this into an account, we designed this study to bridge the gap between parents seeking information and medical doctors who were willing to share their experience on COVID-19 treatment and prevention. A virologist and specialist in the topic of COVID-19 testing was contacted as a consultant. The intervention series was based on online seminars with small groups in different languages due to the vast diversity in population and was augmented by a uniform seminar protocol and implemented quality control. By achieving high effectiveness and reproducibility, this study describes a role model countermeasure of vaccine hesitancy.

## Methods

The core idea in the design of this study was to provide a platform that allows people to profit from the first-hand experience of medical doctors working with COVID-19 patients. Parents who had doubts on whether to vaccinate their children or not had been provided with the possibility of discussing their individual questions with medical doctors. As outlined previously and elaborated in the discussion section, literature review has shown that interventions aiming at educational information of participants show highest success rates when compared to other attempts [16, 19]. Face- to face conversations with special focus on empathic communication including story-telling aspects were identified as highly effective tool to inform and possibly convince people with vaccination skepticism [17, 18, 20, 21]. A previous meta-analysis could gather statistical evidence that face-to- face interventions are among the most efficient tool for educating parents about early childhood vaccination [21]. Individual information sessions conducted by trained medical personal have proven successful in promoting infant vaccination among parent, previously [19]. Adapting the concept of Lemaitre et.al., we made a deliberate effort to minimize the number of questions and avoid using confrontational language while ensuring that we provided careful and informative communication to people [19].

Our study specifically focused on parents of unvaccinated children because 1) there was epidemiological evidence that spread of delta variant was propelled by unvaccinated people below the age of 18 and 2) parents are particularly concerned about the wellbeing of their children. Although Austria has a nationwide, high performing healthcare system, there is no institutional possibility for people to address their individual questions concerning vaccinations.

The intervention was specifically designed for this study. To balance cost efficiency and the possibility of addressing individual questions, we decided to undertake a question-and-answer seminar format in small group sizes. We set the maximum number of participants per seminar to twenty and achieved an average number of 16 participants per seminar. This group size was small enough to allow participants to voice their individual questions (94% stated that they could voice their most important questions). Moreover, group seminars efficiently leverage the impact of time spent by medical experts compared to one-on-one talks.

During the study, there was a widespread public debate about the severity of COVID-19, particularly concerning children and adolescent patients. We recognized this topic to be central to the hazard-benefit debate surrounding SARS-CoV-2 vaccination. With the rise of social media and online videos, there was a growing mistrust in information conveyed through “conventional” TV and news media channels. Our aim was to overcome this mistrust by centering on authentic, first-hand experiences. All medical doctors that participated in the study were selected based on their personal experience with the treatment of COVID-19 or COVID-19 vaccine related adverse events. The onboarding session was used to communicate aims and goals of the initiative and to ensure that the seminars were held in a reproducible structure. Speakers were instructed to preferentially respond to questions by using narrative examples from their own clinical experience. Answers with narrative aspects and concrete examples have proven to be more effective in previous studies [20]. Especially in the context of misinformation bubbles and conspiracy theories, first-hand experiences take an important role in communication of information. Yet, knowledge on statistic results of major studies on vaccine safety profiles, adverse events, and benefit-risk ratios were considered a necessary perquisite for evidence-based argumentation. Hence, this information was included in the onboarding seminar that all the medical doctors attended. Moreover, new and relevant findings were constantly collected, summarized and shared in form of an interactive online document among the staff.

### Participants Intervention Design

Personnel expenses for the seminars were covered by the city government of Vienna, Department of “Wiener Gesundheitsförderung” and by the state government of Lower Austria, department of health and social affairs. Aside from this, the intervention did not receive any other funding and hence was free of conflict of interest. The promotion of seminars was also carried out by the financing institutions, involving school authorities and parents’ associations. Headmasters of all public schools in the provinces of Lower Austria and Vienna received information via email about the seminars. Parents were informed via the school mailing list postings or by announcements on the school’s notice boards. Additionally, the seminars were announced on the social media platforms (Instagram and Facebook) of the medical university of Vienna and the city government of Vienna. Information on what to expect and how to enroll for the seminars was published in German, Turkish, Serbo-Croatian and Arabic. Participants were asked to sign up for one of the seminar dates via email or phone call. Participation was completely free of cost. Registration of participants was organized by an external service provider (P&W phone agency; Vienna, Austria). Lists of participants were kept confidential and not shared with third parties including schoolteachers, school organizational staff, government representatives and commercial companies. Seminar registration and hosting of the online seminars were organized completely independently of school structures so that participants did not have to worry that their participation would declare their opinion on vaccination or the vaccination status of their children.

### Intervention Method

The intervention was designed as an interactive virtual seminar and was conducted via Webex (San Jose, CA, United States). The maximum number of participants was twenty. Each seminar was conducted by one person out of a team of six participating medical doctors. All participating staff members held a degree in medicine and were employed either as medical residents or general practitioners in public hospitals in Vienna at the time of the study. Extensive and personal experiences in clinical treatment and management of COVID-19 patients was a prerequisite for enrollment in the project. On boarding team meetings included explanation, training and observation of seminars. In the onboarding meetings, medical doctors were provided with a document of references and summaries of important studies and guidelines. Moreover, medical doctors were informed that they were not authorized to give individual or medical advice in the course of the seminars. Participants that had questions concerning the medical history of their children were referred to their attending pediatrician. All participating doctors (four male, two female) were below the age of 40 years when the study was conducted. The seminars were offered in four languages: German, Turkish, Serbo-Croatian and Arabic. The team included one Turkish native speaker and one Serbo-Croatian native speaker, both of whom were also fluent in their second mother tongue, German. Seminars in Arabic were planned to be held by a German native speaker with the help of a professional Arabic interpreter. The majority of seminars were scheduled in German. Separate dates were scheduled for Turkish, Serbo- Croatian and Arabic seminars. Links were sent out via email to participants 1 day prior to the scheduled date. The duration of the seminars was scheduled to be approximately 60 min and was split into three parts.

#### Introduction, Framework and Impulse Presentation

Part one (10 min maximum) was made up of a short personal introduction of the expert followed by a structured impulse presentation, using five slides in total (Supplementary Material S1). The first slide showed the general framework of the intervention: preparing the participants for formulating personal questions, clarifying that the seminar was funded by the regional government and did not receive any further funding from third parties, and that the session was not being recorded. Participants were engaged so as to prevent hesitation and be given a platform to voice their questions, concerns or worries regarding any aspects of the SARS CoV-2 pandemic or SARS CoV-2 vaccination. Participants were instructed to adhere to basic rules of discussion: not to interrupt others and not to insult or discriminate against other persons or their opinions. The impulse presentation used three slides to illustrate the current pandemic situation (Supplementary Material S1). All figures and underlying statistics were retrieved from publicly available, government sources of national SARS-CoV-2 infections. The first slide illustrated SARS-CoV2 incidence rates since the genesis of the pandemic. The second slide showed a heatmap depicting incidence rates in age groups over time. Slide three showed the incidence rates over time for the age group of 12–17 years, separated by immunization status: immunized- either vaccination or convalescence; partly immunized-vaccination status longer than 6 months ago, vaccination status less than 7 days ago, confirmed SARS CoV-2 infection longer than 6 months ago; and not immunized. Slide four summarized the current recommendation of the national vaccination commission for the vaccination of children above 4 years of age (Supplementary Material S1).

#### Question and Answer

Part two consisted of a 45 min open ended question and answer (Q & A) and discussion session. Participants were engaged and asked to introduce themselves briefly and switch on their webcam. However, this was not mandatory and one could decide to stay anonymous. Participants were asked to either raise their hand in front of the camera or use the raise-hand function in Webex software if they needed to speak. Wherever possible, experts gave examples from their clinical experience in the treatment of COVID-19 patients. For frequently occurring questions on statistical numbers, we prepared single slides summarizing key statistical points. For example, Supplementary Material S2 was shown when participants specifically asked for statistical numbers on the risk of developing side effects from the vaccine.

#### Feedback and Survey

Finally, for part three of the seminar (5 min), participants were asked to give anonymous feedback via an online survey. The link to the survey was provided via the chat function in Webex software. The survey was conducted using a commercial service provider (SurveyMonkey, Dublin, Ireland) and consisted of six questions. The language of the questions corresponded to the language of the seminar. The questionnaire was translated by the speakers in their mother languages. None of the non-German speakers are licensed or professional translators but speak German on C2 level. No identifying characteristics of the participants were saved.

Question 1 Choose the expert from the list below that has moderated the seminar you attended.

Question 2 Feedback- please rate the following statements (a–e) with either 1) I do not agree, 2) I partly agree, 3) I mostly agree and 4) I agree completely. Statements: (a) I could voice my most important question(s), (b) The overall atmosphere during the seminar was appreciative, (c) The answers were easy to understand, (d) The expert has avoided medical terminology and (e) My most important question(s) concerning COVID-19 vaccination was fully answered.

Question 3 What are/were your biggest concerns about COVID-19 for your children? (Pick one or more); (a) vaccination side effects, (b) development of the vaccine was too fast, (c) distrust towards the pharmaceutical industry, (d) benefit from vaccination too small, (e) long-term side effects (f) restriction of my personal freedom, (g) distrust towards the government and (h) none.

Question 4 Rate your personal willingness to get you child/children COVID-19 vaccinated BEFORE attending this seminar (sliding bar with integer numeric values 0–10; 0 = very low (minimum willingness); 10 = very high (maximum willingness).

Question 5 Rate your personal willingness to get you child/children COVID-19 vaccinated AFTER attending this seminar (sliding bar with integer numeric value 0–10; 0 = very low (minimum willingness); 10 = very high (maximum willingness).

The link to the questionnaire was provided during the last 5 min of each seminar. Hence, it hast to be emphasized that the questionnaire itself was answered only at a single timepoint (after the seminar). Question 4 on vaccination willingness *before* the seminar was therefore answered in hindsight.

Question 6 Any particular praise, criticism or comments? (Free text form)

### Statistical Methods

Answers were evaluated using descriptive statistics. Statistical significance of number of motives in relation to independent motive was calculated using unpaired t-test. Numeric answers to question 4 and 5 were compared using paired t-test. Only entries with numeric answers to both questions were used. Change of numeric vaccination willingness score and vaccination hesitancy motives were compared using Kruskal Wallis test.

## Results

From December 2021 to February 2022, we held 36 seminars with 585 persons attending (16.2 participants per seminar in average). Seminars were attended by 456 participants from the province of Lower Austria and 129 participants from Vienna. Throughout the whole seminar series, we did not report any significant violations of the terms of discussion. None of the seminars had to be aborted ahead of time and none of the participants had to be excluded for disciplinary reasons.

**FIGURE 1:**
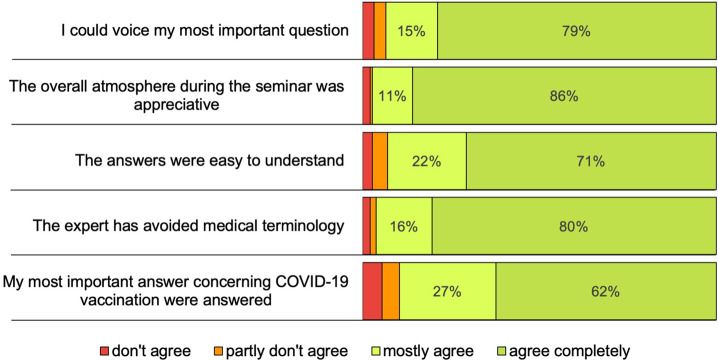
“Results of how participants rated key aspects of the seminar” answers were collected anonymously by online survey during the last minutes of the seminar; *n* = 190 (Austria, 2021).

During the last 5 minutes of each seminar, participants were asked to fill out an anonymous online survey. In total, we received a response rate of 32% (190 survey entries). For question one, participants were asked to choose the person who conducted the seminar they had attended. In question two, participants were asked to quantitatively rate qualitative aspects of the seminar which were identified as key features of this intervention method (Figure 1). Ninety seven percent of participants described the atmosphere during the seminar as either partly or completely appreciative. Ninety four percent felt they had the chance to voice their most important COVID-19-related questions (either partly or completely) during the seminar and 89% either partly or completely agreed that they received a satisfying answer to their questions. Most participants felt that the answers were easy to understand (93% partly or completely agreed) and that the speaker successfully avoided the use of medical jargon (96% partly or completely agreed) (Figure 1).

**FIGURE 2:**
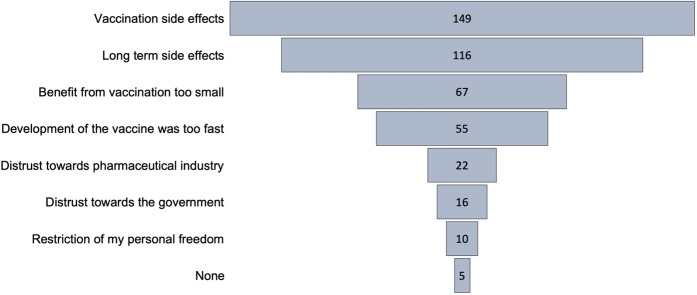
“Motives of Vaccine hesitancy among participants” multiple entries per participant enabled; numbers indicate how often the motive has been selected (Austria, 2021).

Next, people were asked to state their personal motives of vaccination hesitancy by choosing one or more of the given options (Figure 2). Concern on vaccination side effects was the most predominant motive that 78% (*n* = 149) of participants were concerned about. The motive of vaccination side effects was in most cases linked to additional motives (*n* = 124). One hundred and sixteen participants (61%) stated that they were specifically concerned about long term side effects of the vaccination. Thirty five percent (*n* = 67) of total participants were worried that the benefit of vaccination was too small while fifty five percent (29%) participants were concerned that the vaccine development was too fast. Of those, 94% (*n* = 52) were also concerned about the side effects of vaccination. Twenty-two participants (12%) stated that their vaccination hesitancy derives from their lacking trust in pharmaceutical industry. Most of those (*n* = 19) were also concerned about long term side effects. Distrust towards the Austrian government (*n* = 16; 8% of total) was mainly reported by participants who chose at least two other motives of vaccine hesitancy. Restriction in personal freedom was the least frequent cause for vaccine hesitancy (*n* = 10; 5% of total). Five participants selected all the given options and five participants stated that they had no motives for vaccine hesitancy.

**FIGURE 3:**
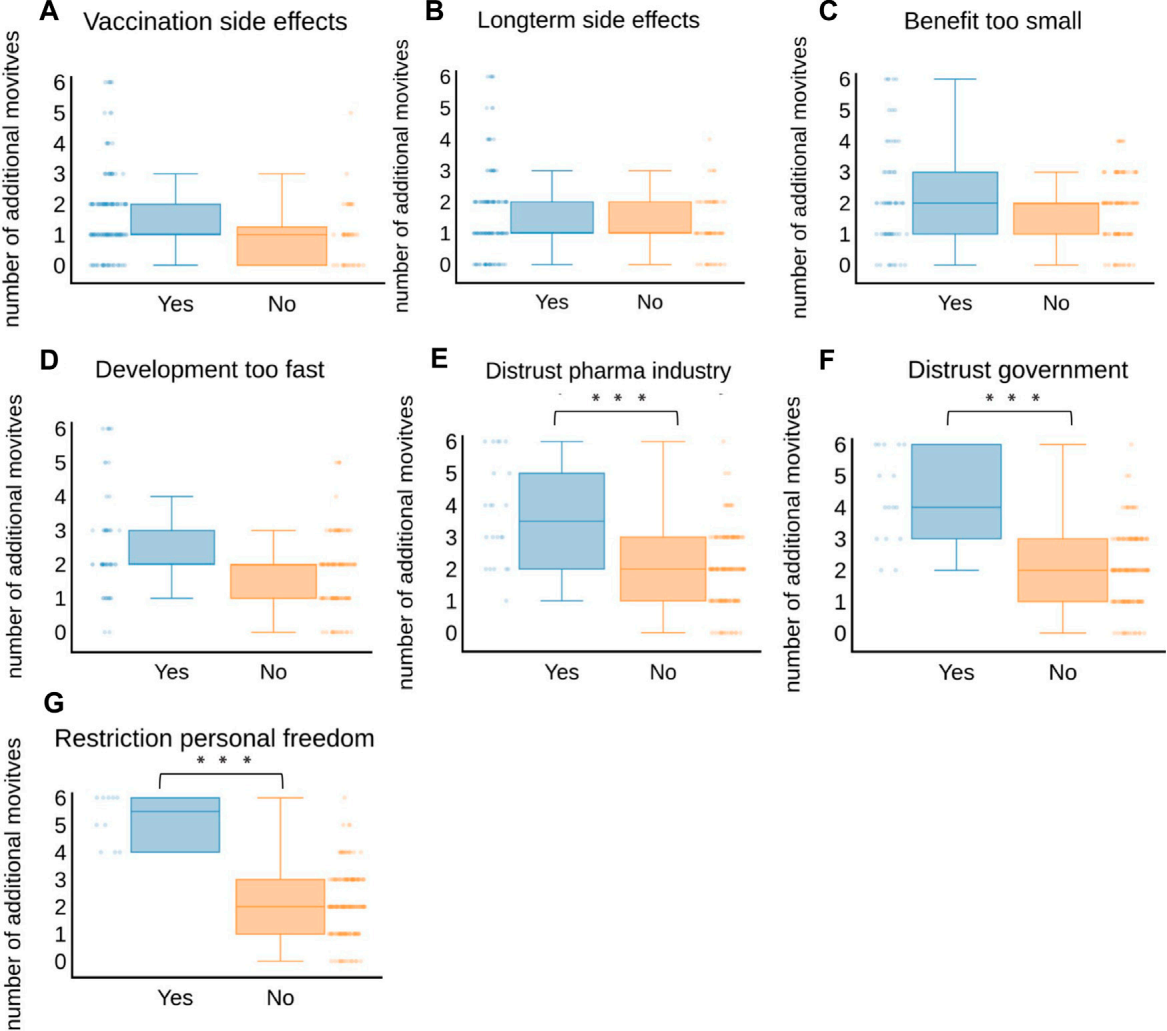
Number of motives of vaccination hesitancy relative to **(A)** vaccination side effects, **(B)** longterm side effects, **(C)** benefit too small, **(D)** development too fast, **(E)** distrust towards pharma industry, **(F)** distrust towards government, **(G)** restriction of personal freedom; Blue plots and bars indicate number of motives of vaccination hesitancy that were selected additionally to the indicated motive; Orange plots and bars indicate total number of motives selected by participants who did not select the indicated motive; statistical significance was calculated using unpaired t-test; *** indicates *p*-value < 0.0001 (Austria, 2021).

Analyzing the number of motives per person, we found that participants who were worried about restriction of their personal freedom or reported distrust in the government or the pharmaceutical industry, were statistically more likely to report additional motives of vaccination hesitancy than others (Figure 3). This observation was most pronounced for the motive of restriction of personal freedom (median 5.5 versus median 2).

**FIGURE 4:**
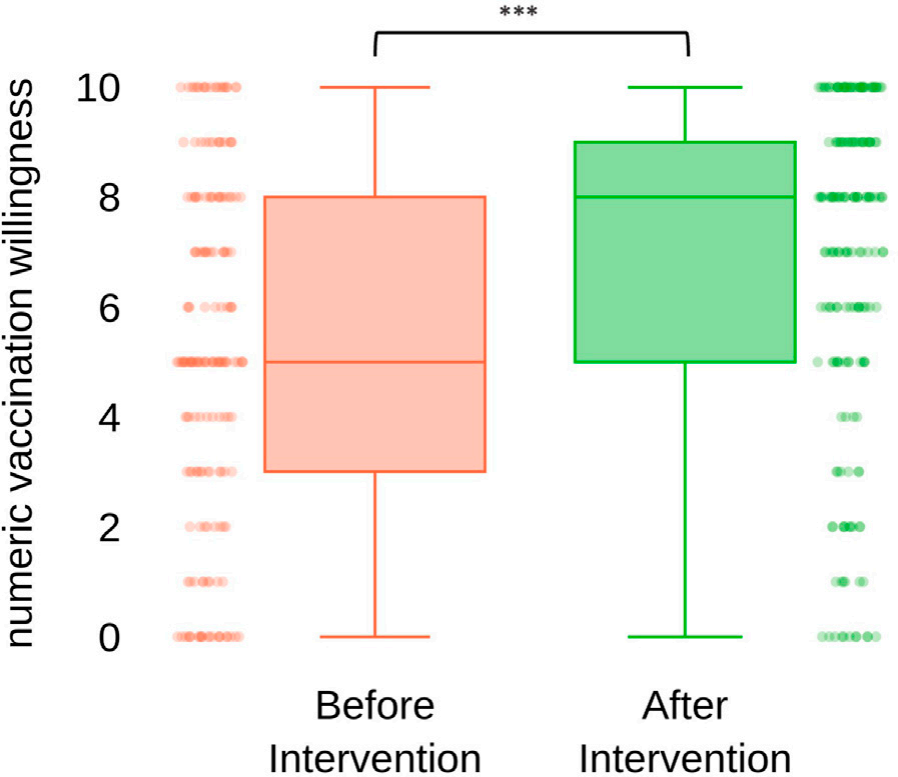
“Quantification of subjective vaccination willingness of participants concerning their children before and after attending the seminar” vaccination willingness was quantified using a sliding bar tool and translated into integer numbers ranging from 0 (minimum) to 10 (maximum); statistical significance was calculated using paired t-test; *p*-value 1.57e-12; xd = 1.5143, *n* = 175 (Austria, 2021).

We tried to quantitatively assess subjective vaccination willingness of participants concerning COVID-19 vaccination of their children. Participants were asked to quantify their subjective vaccination willingness before and after attending the seminar, using a sliding bar tool. The answer was translated into integer numeric values ranging from 0 (not willing to get children COVID-19 vaccinated) to 10 (maximum willingness to get children COVID-19 vaccinated) (Figure 4). In total 175 participants answered this question. Subjective vaccination willingness was significantly increased from a median score of five (before attending the seminar) to eight (after the seminar) (*p*-value 1.57e-12 in paired t-test (*n* = 175; x_d_ = 1.5143). Of all participants with initially very low vaccination willingness (score 0 or 1; *n* = 37)**,** 17 (45%) could be increased by at least one. Of those, eight participants showed an increase of five or more. A single person reported a decrease in vaccination willingness (−2).

**FIGURE 5:**
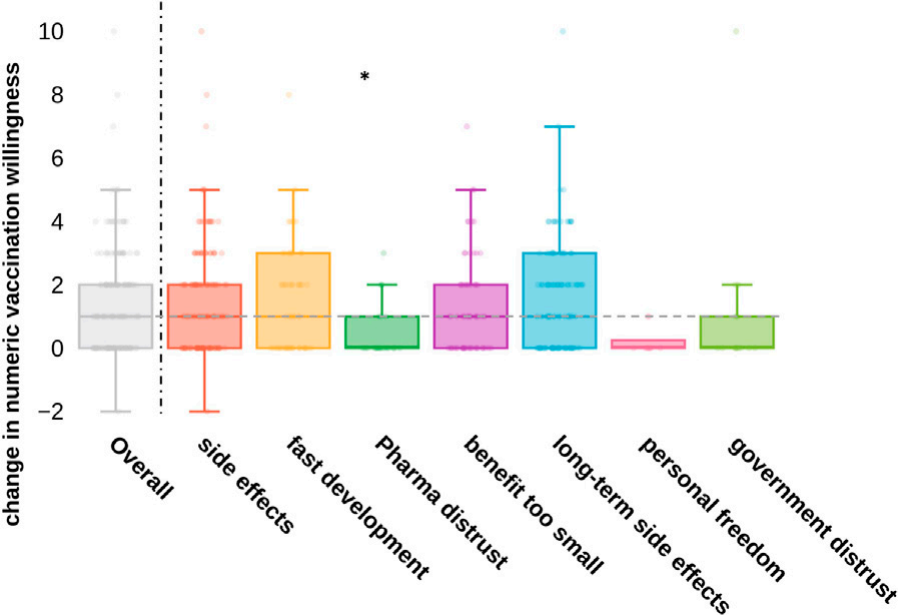
“Change in numeric vaccination willingness grouped by motives of vaccination hesitancy” change in vaccination willingness was generated by calculating the difference of subjective vaccination score before and after the seminar; vertical 551 line divides overall results from results grouped by motives of vaccination hesitancy; selection of multiple motives of vaccination hesitancy per person was enabled; horizontal line indicates overall median change; statistical significance was calculated using unpaired t-test to compare numeric change in vaccination willingness of each group to the remainders; the only statistical significance was found for distrust in the pharmaceutical industry, *p*-value 0.0018 (Austria, 2021).

Although the intervention achieved good success overall, increased rates varied greatly among individuals. Hence, we analyzed score increase rates differences among participants grouped by vaccination hesitancy. We calculated the change in numeric vaccination willingness and grouped results by vaccination hesitancy motives (Figure 5). Median score increase rate for motives “fear of vaccination side effects,” “fear of long term side effects” and “fear of too rapid vaccine development” was in average range.

Participants who reported “distrust of the pharmaceutical industry,” “distrust of the government” and “the feeling of restriction of personal freedom” showed below median increase rates (statistically significant only for pharmaceutical distrust; t-value −2.95 *p*-value 0.0018).

Finally, we evaluate whether there was statistical evidence that the success of the intervention correlated with individual presenters. Calculating the Kruskal-Wallis test on the change in numeric vaccination willingness, we did not find a statistically significant difference between the seminar speakers [χ^2^ (6) = 7.01, *p*-value = 0.320 and mean rank scores of 95.28, 74.92, 53.92, 90.06, 109.42, 104.21 and 93.9; η^2^ = 0.0058].

Question number 6 was formulated as a feedback question where participants could share their personal feedback as free text. The overall feedback was highly positive and in most cases the answers included personal compliments and in single cases personal criticism. All answers were collected and shared with the speakers that held the respective seminars. The feedback was used for internal quality control and as tool for personal improvement for the speakers. However, we decided not to include a record of the answers to this study as in most cases, their content referred to characteristics of individual speakers. An anonymized record of answers to question is available upon request by the corresponding author.

## Discussion

The aim of this study was to address vaccine hesitancy among parents of unvaccinated children. For this purpose, we designed a dialogue-based intervention method that allowed us to address individual concerns of a large number of parents.

The first aim of this study was to address individual concerns, worries or fears on a personal level. One-on- one conversations create an optimal space to answer individual questions comprehensively [18]. However, huge personnel and financial resources would be required to reach many individuals. Hence, we decided to conduct seminars in small groups. Group size was considered as crucial variable at predetermining efficiency and effectiveness of the method [19]. Therefore, we limited the maximum number of participants to 20 persons per seminar. The results of the anonymous online survey confirmed that at this group size, all participants got the chance to voice their most important questions during the 60 min. The duration was exceeded only in exceptional cases. However, larger group sizes could be compensated for by longer seminar duration.

Previous studies have found clear associations between parental vaccination hesitancy and epidemiological characteristics such as gender, educational level, economic status or political identity [22–24]. Detailed epidemiological data were intentionally not collected in this study. This decision was based on initial feedback from the Austrian parents’ association, which reported that some participants expressed concerns about the potential misuse of such information by governmental or school authorities to identify them. The study was conducted during a time of intense polarization, prevalent across Europe but particularly in Germany and Austria. The ongoing debate on COVID vaccination hesitancy has caused significant strain on personal relationships and has led to public polarization, regularly demonstrated through weekly protests in all major cities in Austria. Given this context, our primary focus was to prevent any suspicion and foster a more comfortable atmosphere. Consequently, we excluded all questions pertaining to demographic details. It is essential to acknowledge that the absence of demographic data is one of the significant limitations for conducting a detailed outcome analysis. Nevertheless, we firmly believe that creating a confidential atmosphere is crucial for the successful transfer of educative information and the overall success of this intervention.

*The most important aspect for participants to get a chance to voice their worries, concerns, or questions was an appreciative atmosphere during the seminars.* There were a few attempts of vaccination opposing participants to specifically disrupt the discussion by interrupting others. In these situations, people were reminded that participation was possible only when discussion rules were complied. Throughout all seminars, not a single person had to be forcefully excluded from the meeting.

The second aim of this study was to reach a large number of people, independent of geographic boundaries. We achieved this by organizing the seminars as virtual online conferences. The online format allowed us to offer the intervention simultaneously at two pilot regions in Austria: 1) the densely populated capital city of Vienna [4,600 people per square kilometer (sq.km)] and 2) the rural, regional province of Lower Austria with a mean population density of 89 people per sq.km. Although seminars were promoted identically for both regions (mainly via parental mailing lists of schools and kindergartens), we registered more participants from rural areas than from the capital city of Vienna. Therefore, it can be assumed that there was a larger need for in-person information in rural areas where other information campaigns such as public information counters might have been less accessible than in the densely populated city. Online seminars offer the benefit that individuals can participate independent of their location and save time travelling to and from the seminar. This benefit is more pronounced in less populated areas and might have also contributed to the higher demand in rural provinces. Moreover, organizational costs can be reduced as medical experts do not have to be recruited for each province separately and can host multiple seminars from wherever they are based.

Another benefit of virtual seminars is that participants can maintain different degrees of privacy by choosing not to disclose their full name or live video to the group. This can be of particular importance for polarizing topics such as vaccine hesitancy. However, for this study, almost all participants chose to share their video option and their first names to the audience. The overall acceptance of virtual meetings and online seminars has increased throughout the pandemic. However, a potential limitation of online seminars is that it might discriminate against age groups that are less accustomed to using smartphones or computers. As this intervention specifically focused on parents of children attending public schools or kindergarten, we did not consider this bias to be significant in this age group.

Access to individual information on COVID-19 vaccination was limited by ethnographic affiliation in addition to the geographic limitations mentioned above. Migrant communities with non-German languages as their mother tongue have been shown to suffer from misinformation at a higher degree than the German speaking population [25]. This might be as official vaccination information is translated into various languages, but dissemination of relevant information often takes place via initiatives from the various communities or individual key persons within the communities. It has been shown that migrant communities tend to use media in their mother tongue as a major source for information [26]. To specifically address these communities, we offered seminars in the most common non-German languages of Eastern Austria (Turkish, Serbo-Croatian and Arabic) and promoted the seminars in cooperation with local associations of migrant communities (such as the Turkish Cultural Association Austria “Türkische Kulturgemeinde Österreich”). Despite various efforts, we did not receive any registrations for non-German seminars. It might be that bilingual people of these communities participated in the German lectures. As we decided not to assess information on social or ethnical background of the participants to avoid scepsis, we cannot further address the socio-cultural composition of the overall audience, which again is a limiting factor of our survey.

With more than 580 participants in less than 3 months, we reached out to a large number of individuals within the target group. To evaluate effectiveness of the intervention, we assessed a numeric value of subjective vaccination willingness of the participants regarding their unvaccinated children. The observed increase of a median value of five (before attending the seminar) to eight (after the seminar) emphasizes the significant role that personal conversation has in tackling vaccine hesitancy. While there has been an overwhelming presence of vaccine information in classical and social media, the results of our study underline the importance of information campaigns that focus on individual interest, questions and concerns. Over the course of time, vaccination hesitancy had rather increased despite the measures taken by governments to combat the pandemic. As a result, the call for vaccination was suddenly lumped together with many unpopular and, in retrospect, overreaching policies. In a previous study conducted by Hagood et.al., it was suggested that parents who refuse vaccination of their children in should be categorized into 1) vaccine rejecters: people who reject vaccination on principle, often associated with conspiracy theories, rejecting governments or institutions; 2) vaccine resistants: parents who reject vaccination currently but do consider information and 3) vaccine-hesitant: people who are hesitant and afraid of vaccination, but do not categorically reject vaccination [14]. While people of all three categories have participated in this study, the median vaccination willingness score of five indicates that the average participant did not reject vaccination categorically. Analyzing motives for vaccine hesitancy, we observed that people who mentioned emotional and ideological motives such as “personal freedom restriction,” “lack of trust in the pharmaceutical industry” and generally “lack of trust in the government” reported more additional concerns than others. The same group of participants also showed significantly lower increases in subjective willingness to vaccinate. Hence, this group of people show similar characteristics to the previously described category of vaccine rejectors. Our results confirm the observation of Hagood et.al. that vaccine-rejectors seem to be undirectedly opposed to any topic on vaccination and are not receptive to evidence-based information [14]. However, vaccine rejecters represented only a small minority of participants. Most participants reported concerns on either “vaccination side effects,” “long-term side effects” or “questionable benefit of vaccination.” All these issues were ones that could be easily addressed in discussions with professionals and increase in vaccination willingness confirms that people were susceptive to evidence-based arguments.

This study suffers of several limitations. As discussed previously, this study lacks epidemiological data on participating individuals. This limits the conclusions that can be drawn on the effectiveness of the intervention method for particular subgroups. The intervention was promoted in primary and secondary schools. Hence, the participating parents must have had at least one child at the age 7–18 years. However, we did not collect data on children’s age, gender and number of children with the family. As the recommendation for vaccination of children below the age of 12 changed during the time when this study was carried out, this can be a hidden confounding factor. Arguably, the most significant limitation of this study is the assessment of subjective vaccination willingness at a single timepoint, rather than using a two-timepoint design (before and after the intervention). We believe that this approach helped prevent further polarization among participants before engaging in the discussion and also allowed us to generate paired outcome results while maintaining a straightforward survey design. However, we acknowledge that the single-timepoint assessment may have introduced hind-sight bias. In less emotionally charged settings, employing a questionnaire at the beginning and end of the study would yield less biased analysis and more valid insights. Finally, with an overall response rate of 32% we cannot rule out that the reported results suffer from a self-selection bias. This must be taken into account especially when interpreting the increase in subjective vaccination willingness.

In summary, we could show that structured online seminars conducted in small groups are a highly effective, cost- and time efficient method to tackle vaccine hesitancy of specific target groups. The method is well scalable and easily transferable to other healthcare topics of public concerns. The method is highly scalable and easily applicable to address other healthcare topics that are of public concern. Its use is not confined solely to vaccination campaigns but can extend to various medical education purposes, including topics like risk prevention through lifestyle changes or screening programs. Further studies will determine whether the results obtained for SARS-CoV-2 vaccination hesitancy, as reported here, can be replicated for other subjects.
