# Supplementary figures and images for: Tackling Vaccine Hesitancy and Increasing Vaccine Willingness Among Parents of Unvaccinated Children in Austria
Source: Int J Public Health. 2023 Aug 28;68:1606042. doi: 10.3389/ijph.2023.1606042 (PMC10493290; doi:10.3389/ijph.2023.1606042)

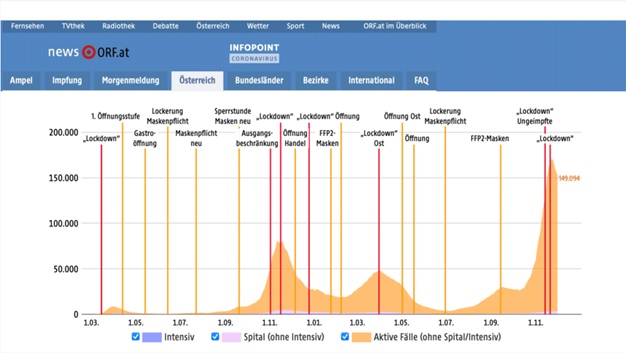

Supplement: Supplementary file 1 [file Image3.JPEG]

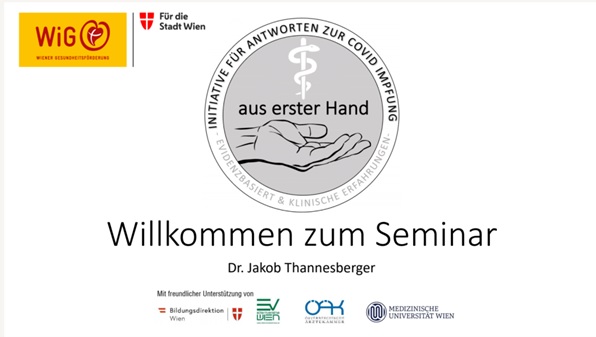

Supplement: Supplementary file 2 [file Image1.JPEG]

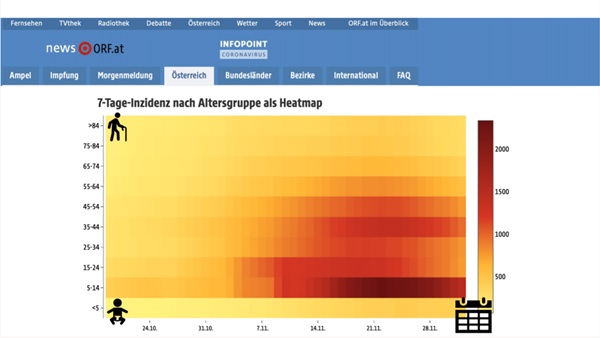

Supplement: Supplementary file 3 [file Image4.JPEG]

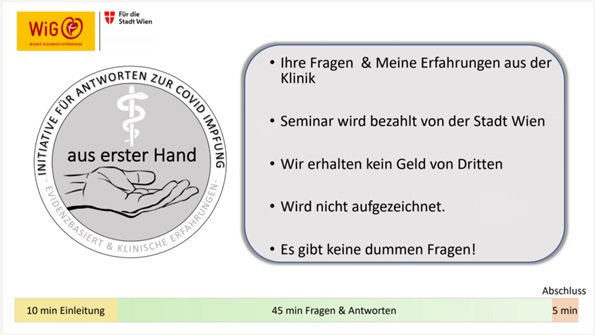

Supplement: Supplementary file 4 [file Image2.JPEG]

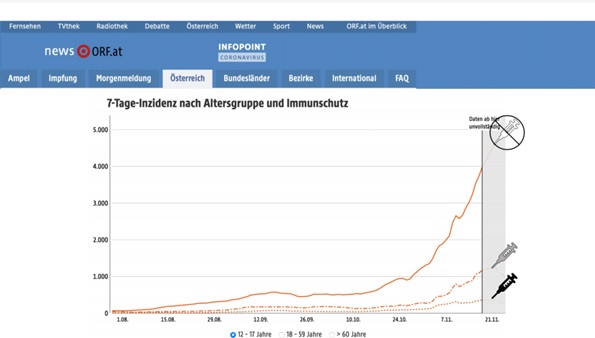

Supplement: Supplementary file 5 [file Image5.JPEG]

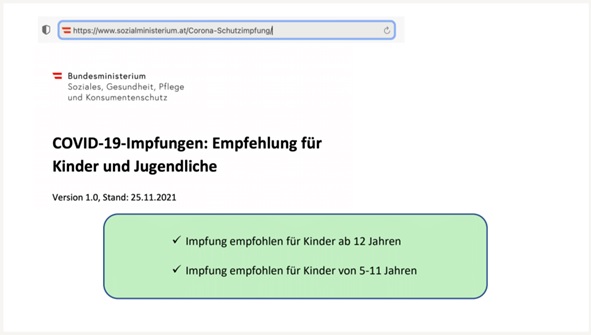

Supplement: Supplementary file 6 [file Image6.JPEG]
